# Delirium caused by topical administration of cyclopentolate for cataract surgery in mild cognitive impairment due to Alzheimer's disease: A case report

**DOI:** 10.1097/MD.0000000000024394

**Published:** 2021-02-26

**Authors:** Yu Yong Shin, Jin San Lee, Key-Chung Park, Hak Young Rhee

**Affiliations:** a Department of Neurology, Naeun Hospital, Incheon; b Department of Neurology, Kyung Hee University College of Medicine, Kyung Hee University Hospital; c Department of Neurology, Kyung Hee University College of Medicine, Kyung Hee University Hospital at Gangdong, Seoul, Republic of Korea.

**Keywords:** Alzheimer's disease, cataract, cyclopentolate, delirium, mild cognitive impairment, mydriatics

## Abstract

**Rationales:**

Cholinergic modification by anticholinergic medication can produce adverse effects in central nervous system (CNS) and cyclopentolate is an antimuscarinic agent widely used for ophthalmologic management. We demonstrate a rare case of hyperactive delirium caused by topical administration of cyclopentolate in a patient with amnestic mild cognitive impairment (MCI) due to Alzheimer's disease (AD).

**Patient concerns:**

A 74-year-old man showed acute confusion after preparation for cataract operation in day surgery clinic. The patient became confused and agitated after instillation of topical cyclopentolate drop into the eye and the symptoms persisted over several hours.

**Diagnosis:**

Previously the patient had been diagnosed with amnestic MCI with the finding of bilateral medial temporal atrophy on brain magnetic resonance imaging. ^18^F-flutemetamol positron emission tomography scan demonstrated multifocal amyloid deposition in the brain.

**Interventions:**

The patient was closely observed with the supportive management.

**Outcomes:**

The patient began to recover 5 h after the onset of symptoms and the cognitive function was reverted to previous state within 24 h.

**Lessons:**

It is well known that several drugs with anticholinergic effects used in perioperative periods make the patients susceptible to delirium, but even the topical administration of cyclopentolate for cataract surgery also produce adverse CNS effects in a vulnerable patient who is diagnosed with MCI due to AD in this case.

## 1 Introduction

The cholinergic system is widely distributed in the brain and plays an important role in cognitive functions such as attention, information processing, memory, and learning. Cholinergic modification by anticholinergic medication can result in anticholinergic syndrome in central nervous system (CNS) that includes cognitive decline, psychosis, seizure, and a variety of neurologic signs. Cyclopentolate is an antimuscarinic agent as mydriatics and cycloplegics and widely used for ophthalmologic examination and surgery with the benefit of rapid onset and recovery. Although CNS adverse event of topical anticholinergic agents is not common, several cases related CNS manifestation after instillation of topical eye drops have been reported.^[1,2]^ We describe a case of hyperactive delirium that developed after the topical application of cyclopentolate for cataract surgery in a patient with amnestic mild cognitive impairment due to Alzheimer's disease (AD).

## 2 Case report

**Figure 1:**
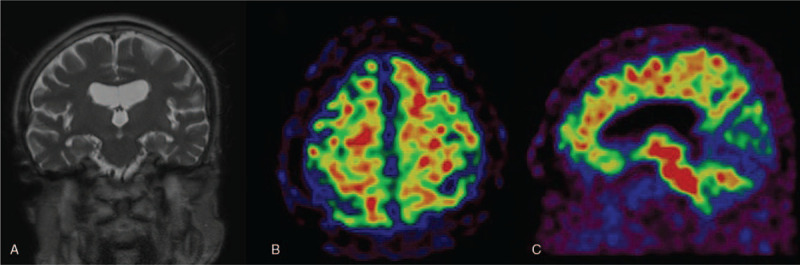
Brain MRI revealed bilateral medial temporal atrophy on coronal plane (A). ^18^F-flutemetamol PET scan demonstrated amyloid accumulation in bilateral frontal lobes (B) and precuneus (C). MRI = magnetic resonance imaging, PET = positron emission tomography.

A 74-year-old, right-handed Asian man with 4 years of education was admitted to ophthalmology clinic for cataract refractive surgery. The patient suffered episodic memory decline over the past 3 years and prior neuropsychological assessment performed 2 years before this visit revealed low performance in memory on the Seoul Verbal Learning Test that was 3%tile score below 1.5 standard deviations from the mean of the age and education-normative values among the Korean population. The patient was not impaired in other cognitive domains. Magnetic resonance imaging (MRI) of the brain showed atrophic changes in the whole brain and bilateral medial temporal lobes and focal chronic infarction in the right cerebellum (Fig. 1A). The patient had been diagnosed with amnestic mild cognitive impairment (MCI) and regularly followed up for the state. The cognitive status was stable over 2 years. The patient had hypertension, diabetes mellitus, and dyslipidemia. Before the cataract operation, one drop of 1.0% of cyclopentolate was administered to patient's eyes. One hour after the instillation of eye drop, the patient got confused and showed abnormal behavior. The patient tried to remove the peripheral venous line and became aggressive to medical staff. The patient was referred to Department of Neurology and the elemental neurological examination revealed no abnormality. Two hours after the event, the Korean version of Mini-Mental State Examination (K-MMSE) was performed and the score was 16 of 30 which had been 26 of 30 on previous test. The patient began to recover 5 h after the onset of symptoms and the cognitive function was reverted to previous state within 24 h. ^18^F-flutemetamol positron emission tomography (PET) scan performed 2 months after the event demonstrated multifocal amyloid deposits in bilateral frontal lobes, the left precuneus, and the right lateral temporal cortex (Fig. 1Band C).

## 3 Discussion

The transient manifestations of the patient which developed after receiving eye drops of cyclopentolate include confusion, agitation, and aggressiveness and lead to diagnosis of hyperactive delirium. The patient had been diagnosed with amnestic MCI which is a syndrome defined by clinical, cognitive, and functional criteria.

It has been well known that anticholinergics has a detrimental effect on cognitive functions. Cholinergic deficiency is recognized as a likely contributing feature to all cause of delirium.^[3]^ The amount of anticholinergic burden in the normal elderly is closely related to cognitive impairment in memory and executive functions and brain atrophy measured by whole brain cortical volume, temporal cortical thickness, and lateral inferior temporal volume.^[4]^ Cyclopentolate is a synthetic anticholinergic agent which produce mydriasis and cycloplegia with the benefit of rapid onset and recovery. Cyclopentolate eyedrops pass readily through nasolacrimal duct and are well absorbed locally as well as systemically via conjunctiva and nasal mucosa.^[5]^ Systemic toxicity includes inappropriate behavior, visual and auditory hallucinations, and cerebellar dysfunction that has been reported mostly in a group of children and adolescents and depends on the dose administered.^[2]^ It has been reported that instillation of several drops of a solution of cyclopentolate produced mental deterioration in an elderly patient with dementia.^[1]^ In this case, we demonstrated the exact neurological status of the patient who suffered mild cognitive decline and showed amyloid accumulation in the brain on PET imaging which suggests underlying pathophysiologic process. Cholinergic deficit can make the elderly with MCI or dementia more vulnerable than the cognitively normal persons to cognitive impairment. Nonetheless, there may be some possible limitations in this study. The causal relationship between delirium and the drug and the exact mechanism may not be clear. The lowest available concentration of the cyclopentolate should be applied for prevention of systemic toxicity especially in patients with cognitive decline.^[2]^

MCI has been known to be transitional state between the cognitive changes of normal aging and dementia but is a heterogeneous clinical condition with several subtypes and multiple etiologies. But the patient showed PET evidence of Aβ deposition in the brain and structural MRI findings for neuronal injury that suggest AD pathophysiological processes in cognitive decline which led to the diagnosis of MCI due to AD. It is well known that several drugs with anticholinergic effects used in perioperative periods make the patients susceptible to delirium, but even the topical administration of cyclopentolate for cataract surgery also produce adverse CNS effects in a vulnerable patient with prodromal AD in this case.

## Author contributions

**Conceptualization:** Hak Young Rhee.

**Data curation:** Yu Yong Shin.

**Writing – original draft:** Yu Yong Shin.

**Writing – review & editing:** Jin San Lee, Key-Chung Park, Hak Young Rhee.
